# The European Paediatric Mycology Network (EPMyN): Towards a Better Understanding and Management of Fungal Infections in Children

**DOI:** 10.1007/s12281-016-0252-7

**Published:** 2016-02-26

**Authors:** Adilia Warris, European Paediatric Mycology Network (EPMyN)*

**Affiliations:** Institute of Medical Sciences, Aberdeen Fungal Group, University of Aberdeen, Foresterhill, Aberdeen, AB25 2ZD UK

**Keywords:** Children, Fungal infection, Neonates, Paediatric mycology, Candidiasis, Aspergillosis, Invasive fungal infections, Antifungals, Epidemiology, Antifungal stewardship, Antifungal guidelines

## Abstract

The European Paediatric Mycology Network (EPMyN) was launched in 2014 to create a European platform for research and education in the field of paediatric mycology. The EPMyN aims to address the lack of paediatric specific evidence and knowledge needed to (1) improve the management and outcome of invasive fungal infections in children and neonates and to (2) enhance and develop paediatric antifungal stewardship programmes.

## Introduction

The European Paediatric Mycology Network (EPMyN) was launched in 2014 to create a European platform for research and education in the field of paediatric mycology. Its mission is to increase the knowledge of the epidemiology and pathogenesis and to improve the management of invasive fungal infections in neonates and children. The detailed objectives of the EPMyN are threefold: (1) to investigate the clinical epidemiology of invasive fungal infections in neonates and children, (2) to investigate new diagnostic and treatment modalities of fungal infections in specific paediatric groups, and (3) to create a forum for educating and training colleagues in the field of paediatric mycology.

The EPMyN is a member of PENTA-ID (www.penta-id.org), a recognised level 1 network for paediatric infectious diseases in Europe by the European Networks of Paediatric Research at the European Medicines Agency (Enpr-EMA). PENTA-ID was developed from the well-established PENTA (Paediatric European Network for Treatment of AIDS) network, originally collaboration between paediatric HIV centres in Europe addressing relevant questions in the field of paediatric HIV. Numerous clinical trials of antiretroviral therapies in children have been successfully performed over more than 20 years and form the basis of current paediatric HIV treatment guidelines [1]. PENTA-ID originates from the need to integrate the expertise and experience gained by PENTA into future studies in other paediatric infectious diseases. The positioning of EPMyN within PENTA-ID will benefit from administrative and legal support, regulatory expertise and extensive expertise in executing multicentre projects and clinical trials. The EPMyN steering group brings together well-known and recognised experts in various aspects of paediatric mycology (neonatologists, paediatric haemato-oncologists, paediatric infectious diseases and immunology specialists, clinical pharmacologists, pharmacist) and embraces a strong combined experience in developing and performing paediatric clinical trials.

### Why

The need for EPMyNcan be found in the unique epidemiology of invasive fungal infections in neonates and children, differences in pharmacokinetics of antifungal agents and usefulness of fungal diagnostic measures compared to adults, and the lack of clinical phase-III trials to assess the efficacy of antifungal agents in the paediatric populations. The lack of paediatric specific evidence results in inappropriate use of diagnostic measurements and antifungals and hampers the development of paediatric antifungal stewardship programmes. Arguments favouring the EPMyN, in addition to the well-established International Paediatric Fungal Network (IPFN) led by colleagues in the USA (www.ipfn.org), are the differences in management strategies (e.g. influence of antifungal prophylaxis, use of diagnostic measures), differences in the fungal epidemiology and in the risk factors associated with the development of invasive fungal infections (e.g. antibiotic prescription policies). A number of colleagues being a member of the EPMyN steering group are actively involved in the IPFN as well and this does reflect the complementarity of these two networks. In addition, developing and executing cross-Atlantic clinical research, taking into account the differences in regulatory, data safety and legal issues between the USA and Europe will enhance future collaborative activities aimed at a better understanding and improved outcome of invasive fungal infections in neonates and children.

### Education and Training

The EPMyN successfully organised its first post-graduate course in Paediatric Mycology on October 12–13, 2015 in Lisbon, Portugal. This post-graduate course was being held in association with the Trends in Medical Mycology 2015, a 2-yearly conference jointly organised by the European Confederation Medical Mycology (ECMM) and the European Organisation for Research and Treatment of Cancer (EORTC). During this 2-day course, the participants were provided with state of the art lectures on the epidemiology, prevention, diagnosis and treatment of invasive fungal infections in neonates, children with primary immunodeficiencies, children with malignancies and those undergoing haematopoietic stem cell transplantation. Interactive case presentations with active involvement of the participants led to vivid discussions and revealed the absence of paediatric specific data to guide clinical decisions.

### Research

Current activities are focussed on collecting the necessary information about the management of invasive fungal infections in children and neonates from a large number of European centres. This information is collected by an electronic survey and the data is captured in the REDCap database. The results of this survey are expected to define specific areas of future research, to highlight gaps in paediatric specific knowledge in clinical mycology and will emphasise difficulties encountered in our daily practice which need to be addressed. In addition, specific surveys are being developed to obtain an enhanced insight in the epidemiology of invasive mould infections in the paediatric population, to describe the experience of fluconazole dosing in neonates with a focus on the use of higher dosages as suggested by recent pharmacokinetic studies [2–4] and to assess the incidence of azole-resistant *Aspergillus fumigatus* in the paediatric population.

Next to these investigator-initiated studies, EPMyN aims to provide a platform for pharmaceutical companies to assist in developing and performing the studies as required in the so-called paediatric investigation plan (PIP) set out by the European Medicines Agency (www.ema.europa.eu). Investigating new antifungals in paediatrics do require specific expertise compared to clinical trials in adults. A PIP need to consider an appropriate medicine’s formulation acceptable for use in children, the need of coverage of all paediatric age groups from birth to adolescence and how to measure its efficacy and side effects. The EPMyN within the PENTA-ID is able to cover those paediatric specific aspects and will provide in expertise needed.

### Guidelines and Antifungal Stewardship

Only recently, professional organisations have noticed that the development of paediatric specific guidelines for the management of invasive fungal infections has been an unmet need and have undertaken a valuable effort to realise the development of paediatric specific guidelines. The European Society Clinical Microbiology and Infectious Diseases (ESCMID)–Fungal Infections Study Group (EFISG) guideline for the management of invasive candidiasis in neonates and children was the first to be published [5•]. In this guideline, both prophylaxis and treatment of invasive candidiasis in both neonates and children are well-addressed. Currently, the guideline for the management of invasive aspergillosis in neonates, children with primary immunodeficiencies and children with cancer is in preparation and its publication is expected in 2016. The executive summary has been presented at the European Conference Clinical Microbiology and Infectious Diseases (ECCMID) in 2014. This guideline will cover recommendations with respect to diagnostic measurements as well as prophylaxis and treatment of invasive aspergillosis. Next to these two fungal disease-oriented guidelines, a guideline for the management of invasive fungal infections in paediatric patients with leukaemia and haematopoietic stem cell transplantation has been published [6•]. This guideline has been developed within the European Conference on Infections in Leukaemia (ECIL) addressing a specific patient population. Although the development of guidelines is not seen as a task per se of the EPMyN, various members of the steering group are and have been actively involved in the preparation of those guidelines.

In complementation of these guidelines, the development of an outline of a paediatric antifungal stewardship programme to be used as a format in individual European countries is under consideration. The need for a paediatric antifungal stewardship programme is directly related to the challenges encountered in the management of invasive fungal infections in neonates and children, the development of antifungal resistance and the high costs of inappropriate antifungal prescriptions. Invasive fungal infections are characterised by unspecific signs and symptoms in already extremely vulnerable children (e.g. premature neonates, children with primary immunodeficiencies or malignancies, and those receiving haematopoietic stem cell or solid organ (e.g. liver, lung) transplants), poor-sensitivity of culture-based microbiologic tests, and the pressure to start treatment early due to the high morbidity and mortality of these infections. Most antifungals in paediatric settings are therefore prescribed for empiric/pre-emptive therapy. Suboptimal dosing of antifungals in neonates and children has been described and may contribute to suboptimal clinical outcomes [7•, 8]. To successfully develop and implement a paediatric AFS programme, evaluation and identification of current gaps in resources and knowledge among healthcare workers involved in the diagnosis and treatment of invasive fungal infections in neonates and children is a key step. This information is currently being collected by us.

### Conclusion

Collaborative efforts in the field of paediatric mycology are critical to improve our knowledge and to facilitate research with the ultimate goal of improving the management and outcome of invasive fungal infections in children and neonates. The EPMyN has taken up the responsibility to provide a platform for research and education in the field of paediatric mycology. The activities undertaken by the EPMyN will facilitate the development of paediatric specific antifungal stewardship programmes built on increased evidence and knowledge.
